# Morphological characteristics and microstructure of kidney stones using synchrotron radiation μCT reveal the mechanism of crystal growth and aggregation in mixed stones

**DOI:** 10.1371/journal.pone.0214003

**Published:** 2019-03-22

**Authors:** Muhammed A. P. Manzoor, Ashish K. Agrawal, Balwant Singh, M. Mujeeburahiman, Punchappady-Devasya Rekha

**Affiliations:** 1 Yenepoya Research Centre, Yenepoya (Deemed to be University), Mangalore, Karnataka, India; 2 Department of Urology, Yenepoya Medical College, Yenepoya (Deemed to be University), Mangalore, Karnataka, India; 3 Technical Physics Division, Bhabha Atomic Research Centre, Indore-Mumbai, India; Institute of Materials Science, GERMANY

## Abstract

Understanding the mechanisms of kidney stone formation, development patterns and associated pathological features are gaining importance due to an increase in the prevalence of the disease and diversity in the presentation of the stone composition. Based on the microstructural characteristics of kidney stones, it may be possible to explain the differences in the pathogenesis of pure and mixed types of stones. In this study, the microstructure and distribution of mineral components of kidney stones of different mineralogy (pure and mixed types) were analyzed. The intact stones removed from patients were investigated using synchrotron radiation X-ray computed microtomography (SR-μCT) and the tomography slice images were reconstructed representing the density and structure distribution at various elevation planes. Infrared (IR) spectroscopes, X-ray diffraction (XRD) and scanning electron microscopy (SEM) were used to confirm the bulk mineral composition in the thin section stones. Observations revealed differences in the micro-morphology of the kidney stones with similar composition in the internal 3-D structure. Calcium oxalate monohydrate stones showed well-organised layering patterns, while uric acid stones showed lower absorption signals with homogenous inner structure. Distinct mineral phases in the mixed types were identified based on the differential absorption rates. The 3-D quantitative analysis of internal porosity and spatial variation between nine different types of stones were compared. The diversity among the microstructure of similar and different types of stones shows that the stone formation is complex and may be governed by both physiological and micro-environmental factors. These factors may predispose a few towards crystal aggregation and stone growth, while, in others the crystals may not establish stable attachment and/or growth.

## Introduction

Kidney stone disease is caused by deposition of mineralized crystals in the renal calyces and pelvis, and these depositions transform into stone(s) in a series of events. The stones formed are managed with surgical procedures or medical interventions; however, prevention of recurrence requires an understanding of the stone composition for medical management that is specific for the stone mineral type [1, 2]. Crystal retention and growth are governed by various factors such as hyaluronic acid, osteopontin, composition of the renal tubular epithelial cell surfaces along with superstauration [3, 4]. However, differences in the interplay of these factors and other intrinsic physiological and metabolic factors may govern the crystallization process and determine the compositions [5].

The microstructure of the stone matrix varies among the stone types and is determined by the mineral composition. The compositional analysis reveals the mineral types responsible for the stone formation, and the ultra-structural investigation of kidney stone matrix provide additional details that are crucial links to the pathogenesis [6]. Certain stones especially composed of calcium oxalate are attached to ‘plaque’, while others often form in free solution in the renal collection system. Under each of these conditions the nucleation and aggregation process differ significantly allowing the stone to aggregate and grow under different conditions leading to heterogeneity in the physical and chemical architecture. During the progression of stone genesis, extensive and repeated dissolution can also occur and may alter the stone matrix at ultra-structural level [7]. The renal inflammatory injury induced by cell-crystal reaction plays an important role in the formation of intra-renal calcium oxalate crystals [8].

The diversity in the lithogenesis process itself can significantly influence the stone internal structure and composition. Further, the mineral distribution pattern in the stone matrix is defined by the mineral accumulation process during the crystal growth that is governed by the availability and or super-saturation of the species. Various advanced techniques are used to study the kidney stone composition and ultrastructure [7, 9]. In a study, detailed characterization of Randall’s plaque using μCT provided details on the mechanism of stone formation; especially the role of apatite in stone formation within the interstitium as a denser portion present within the plaque [9]. A high diversity of mixed stones among the patients indicates that the nucleation process can be random events that can favour the co-aggregation of other urine crystals and facilitate the stone retention. Hence, it is important to understand the relative distribution of the different mineral types in the kidney stones using technology that is more precise and accurate.

Imaging using synchrotron radiation X-ray computed microtomography (SR-μCT) due to higher photon flux in parallel beam morphology as compared with conventional μCT provides higher resolution details in 3-D [10]. Kaiser et al. [11] initially used SR-μCT to study the microstructure and mineralogy of kidney stone with 3-D features and internal composition of kidney stones. This study showed the presence of apatite as concentric and continuous layers in certain stones especially in calcium oxalate stones. However, the study was limited to fragmented kidney stones and could not represent the actual growth pattern characteristics. Hence in this study, we employed SR-μCT to study the texture, mineral deposition pattern, 3-D quantitative analysis of porosity and spatial variation of intact kidney stones representing major mineral types.

## Methods

### Ethics statement

All the procedures involving human participants were approved by the Yenepoya University Institutional Ethics Committee (YUEC.022/16) and Yenepoya Research Centre Scientific Review Board (YRCSRB034/17). The study was performed in accordance with the ethical standards of the institutional and/or national research committee and with the 1964 Helsinki declaration and written informed consent was obtained from all the participants.

### Sample and specimen

Surgically removed kidney stones (n = 22) were collected from the patients with symptomatic stone diseases. All the patients underwent baseline assessment, including detailed medical history and complete blood and urine analysis and non-contrast computerized tomography (NCCT). Each patient had his or her kidney stone(s) removed following the common clinical practice (laparoscopic, percutaneous nephrolithotomy or open stone surgery). The intact stones removed were washed and dried prior to SR-μCT investigations.

### Analytical procedures used in the blood analysis

Blood (5 mL) was collected in vacutainer from each patient using Ethylene diamine tetraacetic acid as a preservative. Blood sugar was estimated by glucose oxidase-hydrogen peroxide (Trinder) method. Serum calcium and electrolytes such as sodium, potassium, and chloride were estimated by Direct-ISE (VITROS 5600 Integrated System Ortho-Clinical Diagnostics NJ, US). ESR was assessed by the photometry method. The 24 h urine pH was measured using a digital pH meter.

### SR-μCT scans and 3-D reconstruction

The experiments were performed at the X-ray imaging beamline (BL-04) on Indus-2 synchrotron source at Raja Ramanna Centre for Advanced Technology, (RRCAT, India). The experimental setup for X-ray μCT consisted of motorized precision translation stages x, y and z and a rotating stage [12, 13]. For data acquisition, the kidney stone samples were mounted over the sample holding chuck. The energy of the incident X-ray beam was optimized for the samples depending on their thicknesses and approximate composition (24–30 keV). The effective voxel size was 2.25 micron. The samples were rotated about their axis in the angular range of 0–180° with step size 0.2° and a total of 901 radiographic projections were collected for each sample.

### Image reconstruction and analysis

Tomography slice images were reconstructed using filtered back projection method to represent the density and structure distribution at various elevation planes in the samples. The cross-sectional slice images were stacked together, volume rendered to show stone microstructure in 3-D and to highlight the features. Quantitative analysis of the SR-μCT data on grey values and porosity variations was carried out using ImageJ software. Porosity was calculated from the images using the Otsu method of threshold followed by noise removal using a median filter [14]. Grey value calculation was done using average grey value for every 10 slices while propagating from top to bottom.

### Compositional analysis using XRD, FT-IR spectroscopy, and FESEM

Following the SR-μCT studies, the stone was subjected to chemical composition analysis using XRD, FT-IR spectroscopy and Field Emission Scanning Electron Microscopy (FESEM). The XRD patterns were recorded with a laboratory diffractometer (Rigaku MiniFlex 600) using Cu-Kα radiation (λ = 1.5406 Å). The diffraction patterns were registered within the 2θ angle range from 10 to 50° and from the diffraction pattern the crystalline phases were identified [15, 16]. The mineral compositions were assessed using ATR-FTIR (Shimadzu IR Prestige-21), in the frequency range of 4000–400 cm^-1^ at 4 cm^-1^ resolution [17, 18]. The FESEM was used to study the microstructure and morphology (Carl Zeiss, Germany).

### Statistical analyses

Statistical analysis was performed using SPSS, Version 22.0. (IBM Corp). Results of categorical data were summarized using frequencies and percentages. Continuous variables were reported as mean ± standard deviation. One-way ANOVA was used to compare means of quantitative variables across the stone types. Significance tests were two-sided and the value of *p*<0.05 was considered statistically significant.

## Results

**Fig 1:**
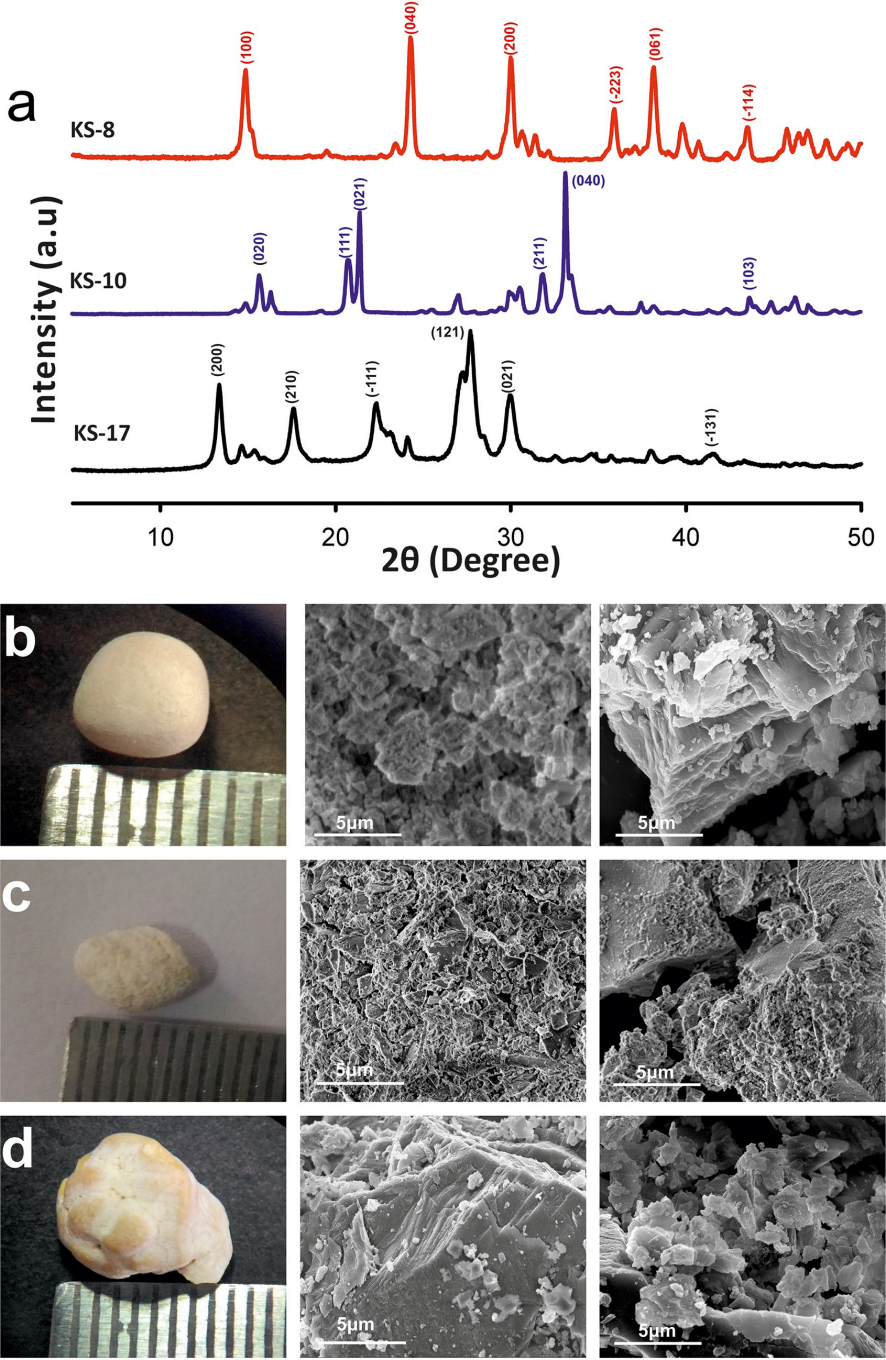
Representative XRD and FESEM images of kidney stones used in the present study. (a) KS-8 and KS-10 COM and struvite crystals showing highest peak intensity at (040) plane. KS-17 Uric acid crystal showing preferred texture growth along (121) plane. (b-d) Pure stones viewed by conventional photography and FESEM. (b) COM appeared as reticulate type of appearance and boundaries of small crystallites. (c) Struvite crystals showed small crystals with hemimorphic morphology. (d) Uric acid crystals showed agglomerates of plate like crystals with clusters of small crystals (scale 10 μm).

Among all the 22 stones tested, 16 (72.7%) were derived from males, and 6 (27.3%) were from females. Summary of patient demographics is shown in Table 1. The stones represented 9 mineral types namely; calcium oxalate monohydrate (COM), uric acid, struvite, COM-apatite mixed, struvite-apatite mixed, COM-uric acid mixed, COM-calcium oxalate dihydrate (COD) mixed, COM-COD-apatite and struvite-COM-apatite mixed. Majority of the patients showed blood biochemistries in the normal ranges; the clinical characteristics such as blood and urine biochemistry are given in S1 Table. The XRD pattern and FESEM images of representative samples are shown in Fig 1.

**Table 1:** Patient demographics and stone mineral composition.

Sample ID	Age	Sex	Location	Side	Recurrence	Mineral
KS-1, KS-2	40	M	Kidney	Right	No	COM
KS-3	64	M	Kidney	Right	No	Uric acid
KS-4	31	F	Kidney	Right	No	Struvite- apatite mixed
KS-5	39	F	PUJ*	Right	No	COM-uric acid mixed
KS-6, KS-8 KS-16	34	M	Kidney	Bilateral	No	COM
KS-7	64	M	PUJ	Right	No	Struvite—COM-apatite mixed
KS-9	37	M	Kidney	Left	No	COM-apatite mixed
KS-10, KS-11	53	F	Kidney	Left	Yes	Struvite
KS-12	50	M	Kidney	Bilateral	Yes	COM-COD-apatite
KS-13	65	F	Kidney	Right	Yes	COM -uric acid mixed
KS-14	34	M	Ureter	Right	No	COM-COD mixed
KS-15	65	M	kidney	Left	Yes	Struvite-apatite mixed
KS-17	24	M	Ureter	Right	No	Uric acid
KS-18, KS-19	50	M	PUJ	Right	No	COM-COD-apatite mixed
KS-20	42	M	Ureter	Right	Yes	COM-COD-apatite mixed
KS-21	60	F	Kidney	Left	No	Struvite
KS-22	34	M	Kidney	Left	No	COM-apatite mixed

*PUJ- Pelvi-ureteric junction, COM-Calcium oxalate monohydrate, COD-Calcium oxalate dihydrate

### Micro-tomography of pure stone types: SR- μCT

**Fig 2:**
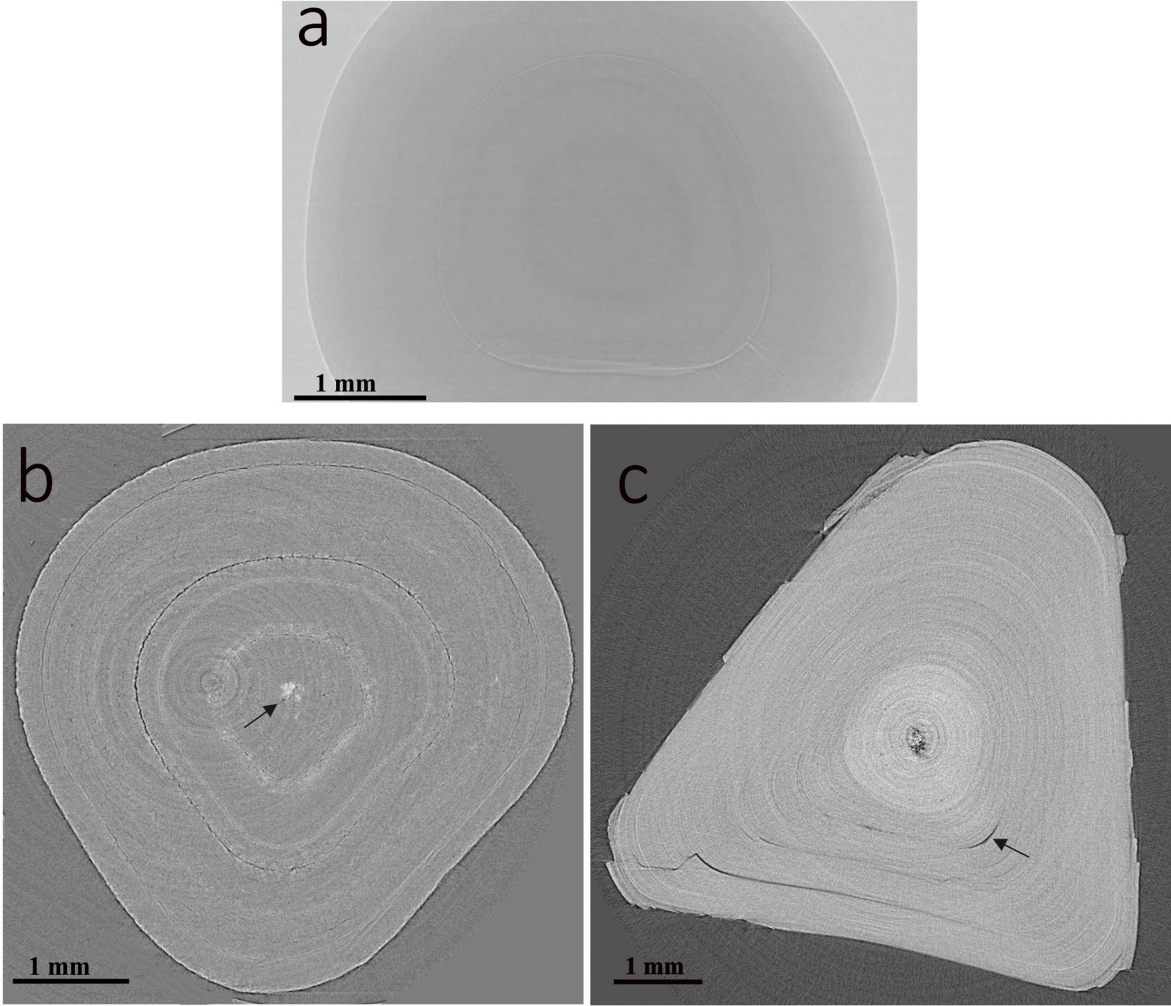
Pure COM stone micro-morphology. (a) A reconstructed μCT slice showing concentric layers of organization. (b) A typical SR-μCT image slice showing visible, radial pattern with concentric layers of organization showing a prominent ring artefact. Arrow mark shows central denser region of high X-ray attenuation values consistent with apatite nucleus. (c) SR-μCT slice showing multiple layers of organisation. Arrow mark shows the presence of visible internal cracks.

**Fig 3:**
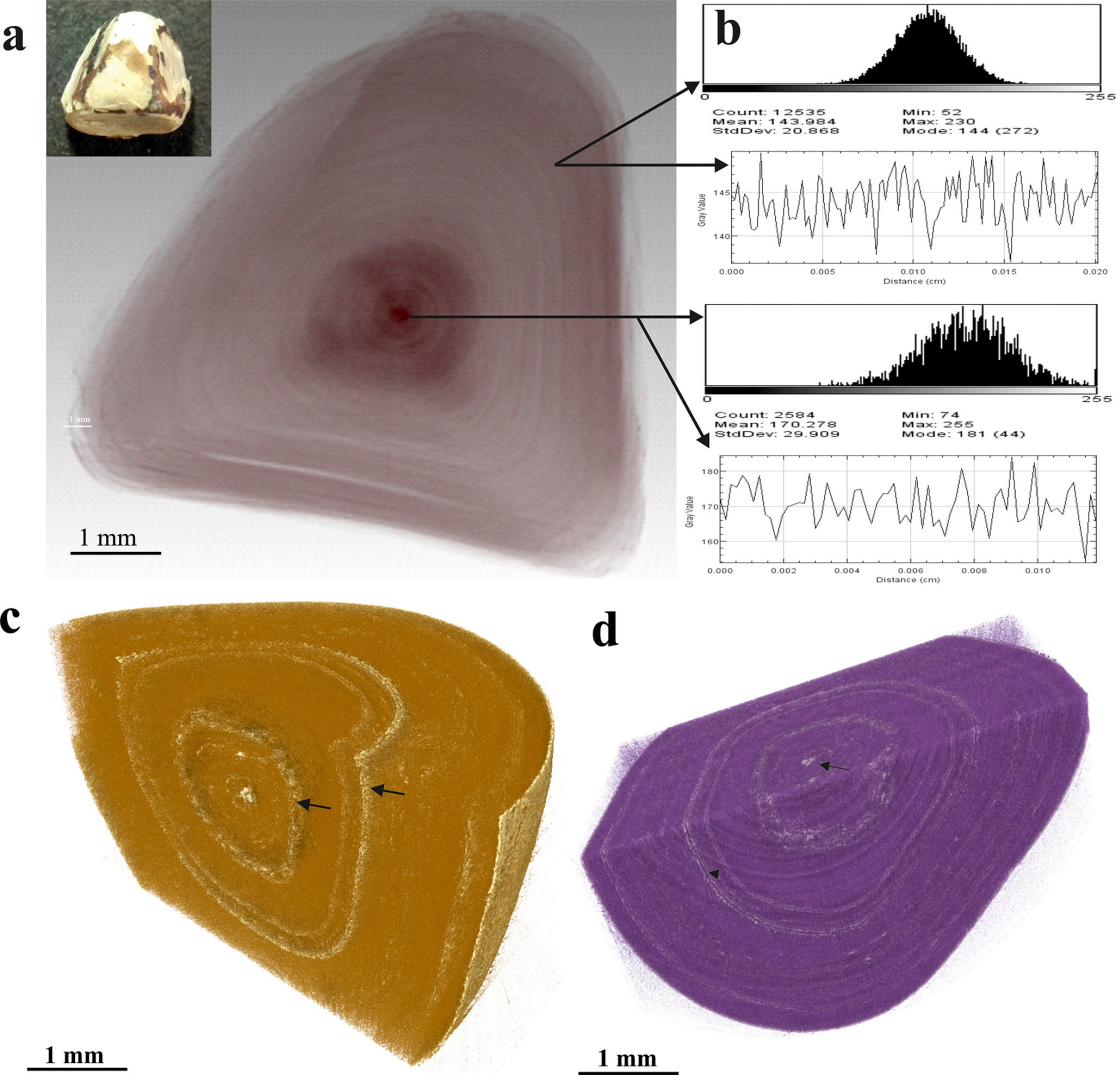
Micro-morphology of COM showing tomographic slice image and the internal 3-D morphology. (a) Maximum-intensity projection of the stone that displays the internal 3-D morphology. Inset showing the conventional photography. (b) Histogram and profile lines measured for outer layer and internal apatite nucleus showing the difference of density in the two regions. (c & d) 3-D reconstruction of the stone that displays the internal 3-D morphology showing a central denser nucleation point and the presence of apatite nucleus. Arrow marks show occasional layers of apatite laid down in the COM.

The COM stones exhibited visible, shell-like texture with a radial pattern and concentric layers of organization. The images with mineral density and grey-scale architecture are given in Fig 2. The central denser region of high X-ray attenuation values was consistent with the core of apatite nucleus surrounded by well organized outer layers. COM stones showed multiple layers of organisation with visible internal cracks (Fig 2B & 2C). The porosity was 0.23% (S2 Table). Similar microstructure in different stones derived from the same patient was observed. However, the microstructure varied in samples derived from different patients (S1 Fig). Fig 3 shows a reconstructed tomographic slice image, the internal 3-D morphology and the histograms indicate the differences in the density between the apatite core and outer layers. The tomographic slice image of different sections of the stones showing mineral precipitation and accumulation of minerals at different elevation planes is given in S2 Fig.

**Fig 4:**
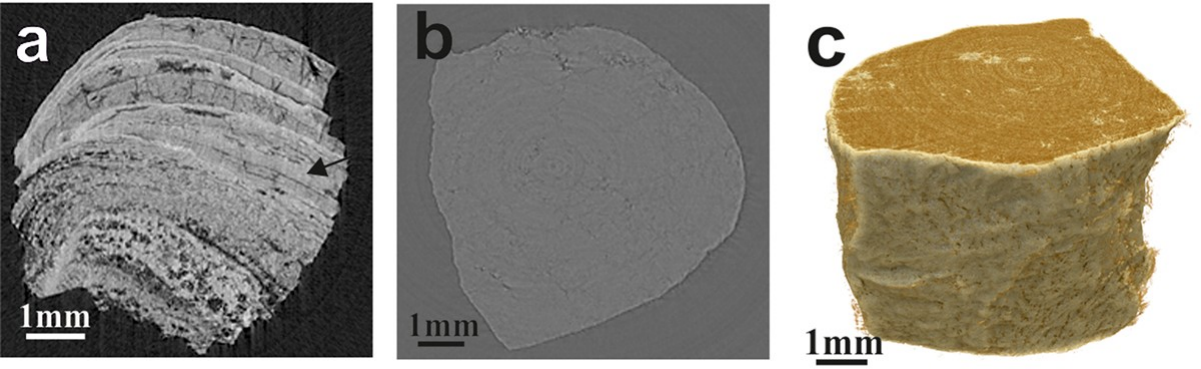
Micro-morphology of pure stone types. (a) SR-μCT image slice of struvite stone showing a pattern of two separate layers with void spaces, micro and macro pores (arrow). The thin white lines showing the apatite. (b) SR-μCT image slice of uric acid stone showing homogenous texture. (c) 3-D surface rendering with slice across the image stack of uric acid stone.

The struvite stones consisted of layered pattern and exhibited void spaces with cracks inwards from the outer surfaces (Fig 4A). The apatite content in the struvite specimen was conspicuous with increased X-ray absorption. The porosity of struvite stones was 7.9% (S2 Table). The uric acid stones showed lower absorption signals compared to other stone types. These stones were less porous (4.36%) and the inner structure displayed multinucleation points with homogenous texture (Fig 4B). However, the outer surface of the uric acid stones showed heterogeneous regions with distinct surface roughness containing small cavities and sharp edges (Fig 4C).

### Micro-tomography of mixed stone types

**Fig 5:**
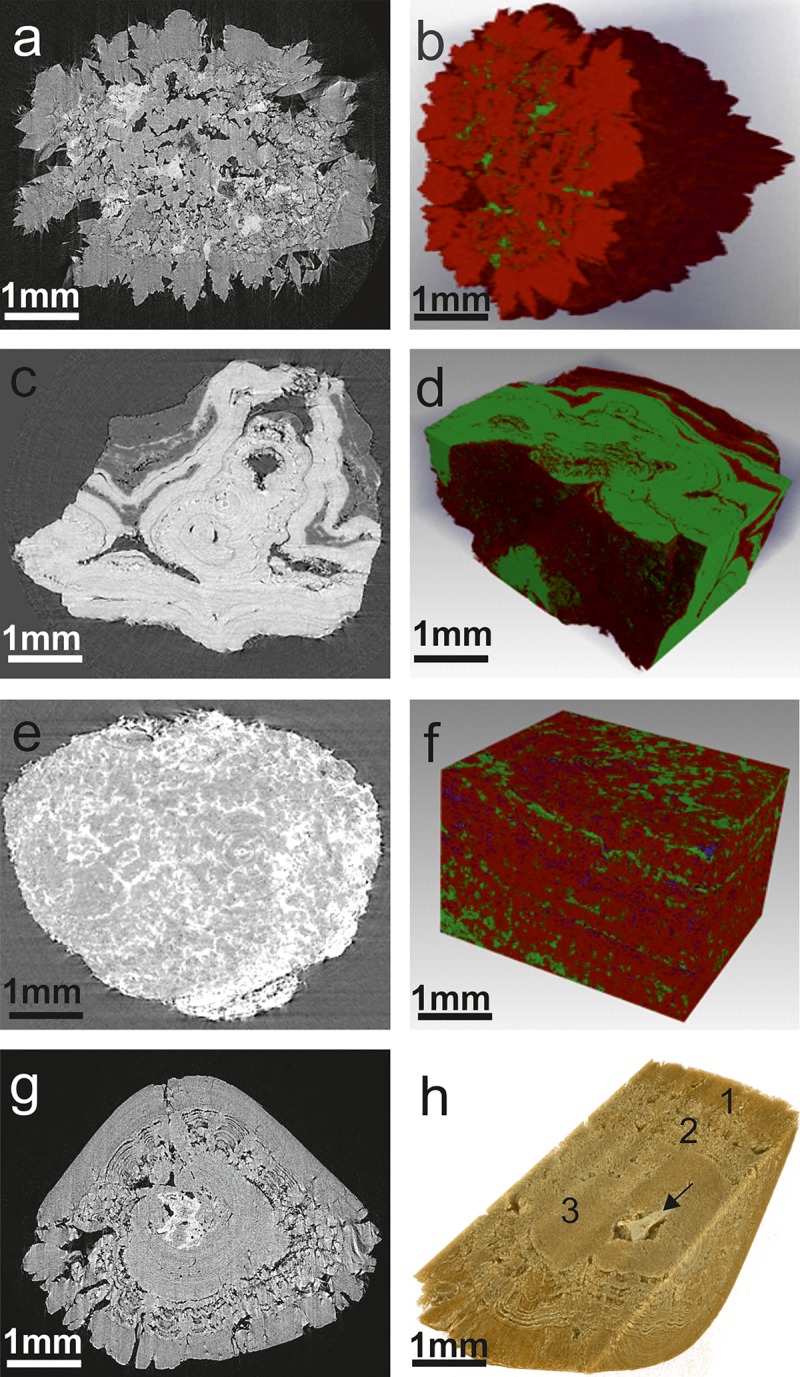
Micro-morphology and 3-D reconstruction of the representative mixed stone types. (a & b) COD-apatite mixed stones. Apatite found along with COD in an unorganized manner. The apatite mixed stones showed relatively high absorption signals and articles of embedded crystals (green). (c & d) COM-uric acid mixed stones. The COM crystal components having relatively higher absorption (green) are directly inverse to the relatively weak absorption observed in uric acid crystals (red). (e & f) Struvite-apatite mixed showing two mineral types by distinct X-ray attenuation; struvite (red), apatite (or carbapatite) (green) and porous air cavities (blue). (g & h) COD-COM-apatite mixed. These stones exhibited visible internal micro and macro pores and showed three distinct layers with varying density and porosity. Arrow indicates apatite nucleus.

The mixed types of stones comprised a more variable crystalline growth pattern, having more irregular and diverse shape and structure (Fig 5). The mixed stones with apatite and COM presented unorganized distribution of the mineral components in the stone matrix. All the mixed stones having apatite component showed relatively higher absorption signals and particles of apatite crystals (Fig 5A & 5B). The COM-uric acid mixed stones displayed intricate structure,calcium oxalate crystal components having relatively higher absorption and weak scattering signal and the opposite was observed in uric acid crystals (Fig 5C & 5D). These stones exhibited a relatively visible radial pattern of core structures. The struvite-apatite mixed stones showed comparatively higher X-ray attenuation compared to other stone types (Fig 5E & 5F). The COM-COD-apatite system, the calcium oxalate component exhibited uniform microtomography, while the apatite showed a relatively denser structure and X-ray attenuation (Fig 5G & 5H). In the case of COM-COD and struvite-COM-apatite mixed stones, apatite showed a relatively denser structure compared to calcium oxalate (S3 Fig).

### Porosity and grey value variation in pure and mixed stone types

Porosity was calculated based on the percentage area of pores in the samples after binarization using standard methods. We found a distinct variation in the porosity among different types of kidney stones as well as stones from different patients in the same group (S2 Table). The purity of the stones was evident from the uniformity in the shade of grey distribution in segmented tomography slices. Among the stone types, the least porosity was found in pure stones and highest porosity was observed in mixed stones (S2 Table)

## Discussion

The *ex vivo* imaging is often used to build data on stones of different morphologies obtained from diverse patient populations. In this study, we compared 22 kidney stones with 9 different compositions (3 pure and 6 mixed types) using SR-μCT. We observed variation in the micro-tomography among the similar type of stones as well as among different composition. The mixed composition stones displayed high heterogeneity in the 3-D internal morphology. This heterogeneity may be due to the fact that crystallization is influenced by many factors like the concentration of ions, their chelators and ionic strength [19]. Similarly, we observed variations in the porosity among different types of kidney stones as well as stones from different patients in the same group that may indicate the mineral concentration or rate of crystal accumulation during the growth. Highly porous stones like COD and struvite are susceptible to shock wave lithotripsy (SWL) compared with less porous stones such as COM. The porosity variation among mixed type stones may pose a challenge during SWL.

Calcium oxalate, struvite and apatite stones exhibit relatively high absorption signals, while directly inverse observations were found in case of the uric acid stone, which is in accordance with previously described chemical and morphological stone properties [20]. Keeping the operational conditions similar (conditions of source size, beam energy and magnification) for the analysis of all stone types in the same facility the variations observed in the grey distribution is attributed to the inherent mineralogical and morphological variations of different types of the stones. We used the original greyscale for the identification of different stones and this imaging system can be correlated to non-contrast helical CT in human [21].

Pure COM stones exhibited a radial pattern and concentric layers of organization with apatite in the nidus [22, 23]. Most of the calcium oxalate stones generally grow from Randall’s plaque having apatite core. The reconstructed tomographic slice image of pure COM suggests that the layers could have been formed by organic material being laid down first and crystals forming within the matrix [24]. Moreover, the urinary constituents like citrate and osteopontin can involve in shape modification and modulation of crystal habits as well the rate of crystal aggregation [25]. It has been shown that the formation of COD crystals is mostly associated with a high urinary calcium concentration and COM crystallization is associated with high oxalate concentration, thus supporting the concept of calcium-dependence of COD and of oxalate-dependence of COM [26].

Uric acid stones are known to grow in a layer-wise manner as concentric rings around a crystallite core [22, 27]; however, in our cases, it was not observed. It has been shown in earlier reports that the crystal size of uric acid stones is significantly different between male and female patients [28]. Previous studies have highlighted the difficulty in interpretation of struvite stone by CT mainly due to the presence of other mineral components [29]. However, struvite can be distinguished in μCT from other stone types due to its characteristic internal structure and radiation attenuation properties. According to a previous investigation, bacterial imprints can also appear on kidney stones with small nanocrystals, such as carbonated apatite than with large nanocrystals, such as struvite [30].

Among the kidney stones, a relative high proportion and diversity of mixed type stones are now commonly reported [31]. The mechanism and sequence of events during the mixed stone growth is highly diverse and yet to be fully explored. The mixed mineral phases present distinct radiographic attenuation values corresponding to each mineral composition in the mixed stone matrix and this enables the visualization of crystal structures and stone mineralogy [11]. For example, in COM-apatite mixed stones, the apatite found along with COM was arranged in an unorganized manner unlike the apatite core in the pure COM. Though, the crystallization process cannot be easily outlined; it may be formed by multiple apatite nucleation interrupting the continuous COM crystal growth resulting in this mixed morphology. It can be possible to explain with the theory of nascent crystal growth from the ductal plugs, with aggressive growth of two different minerals. The process may be governed by the urinary pH, and rate of calcium and oxalate excretion [5, 32]. Due to the chemical interaction and relative concentration of mineral species distributed in the urine, the stone formation can be modified with the incorporation of other minerals present in the urine to a mixed-component complex stone. Studies have shown the co-aggregation effect of amorphous calcium phosphate to promote the formation of large calcium oxalate complexes forming COM stable nuclei. And these clusters aggregate around calcium phosphate that provides multiple sites for nucleation and growth, resulting in multiple COM crystals encapsulating the calcium phosphate [33]. This may be a long process mediated by the biochemical characteristics of the urine and physiological process of the patient. Similar observations were also made showing large porous non-homogenous regions throughout the samples with fragmented particles accumulated in micro-CT by Miller et al. [34]. The presence of uric acid in calcium oxalate stones has been commonly observed and may be a result of the similar crystal lattices present in both crystal types. It is also shown that uric acid binding proteins that bind to uric acid can act as a bridge to bind calcium oxalate to uric acid during the stone formation [35].

Based on the 3-D distribution pattern of the mixed composition in the stone matrix, it is evident that incorporation of different mineral compositions may be dependent on the chemical interaction, changes in the pH and supersaturation of species. The relative distribution of the species may determine the kinetics and thermodynamic aspects of the crystallization process. Mass transfer of a solute from supersaturated liquid solution to a solid crystalline and mixing, also have an effect on crystal purity and morphology. During the initial aggregation stages, the stone exterior may exhibit solute exchange allowing the binding of biological and non-biological entities that can attract various stone constituents, and help in the adherence of smaller crystals to form aggregates.This process will help in aggregation and/or binding of crystal phases. The retained crystals will eventually continue to accumulate the mineral with a periodic interruption in the process by the precipitation of organic material leading to complex structures. Some of the accumulated minerals may get degraded due to dissolution and form micro-porous structures. Similarly, the organic components over the long retention time may get degraded and lost leading to the porous structure. The minerals in the stone may also interact with urine constituents resulting in concentration or leaching as represented by density variation in the stone microstructure. These processes may also encompass the phenomenon of secondary nucleation of newly formed crystals on the surface of those already formed. Our SR-μCT images confirm the law of superposition (i.e., older layers at the bottom and younger layers at the top) in the mixed stones [7]. The porosity in these stones can vary depending on the distribution of other elements. Hence, SR-μCT can be used to easily distinguish the compositions of each mineral and quantitatively calculate the percentages of the total volume of the stone samples based on the radiographic attenuation values of each segmented planes. These parameters can be used to establish the stone growth rate and estimate the age of the stone. These require both *in vitro* and *in vivo* parallel studies in comparison with clinically received samples.

## Conclusion

SR-μCT technique holds potential for better analysis of kidney stones especially their micro-structure, 3-D quantitative analysis of porosity and its spatial variation, thereby providing useful information for the understanding the disease. The micro-morphology of the kidney stones of similar composition showed diversity in the internal 3-D structure indicating different mechanisms of stone initiation and growth. Mixed stones can be formed due to the interaction between the two mineral phases or may also be influenced by the biological residues released due to the damage caused by the stone retention in the tissues. With further efforts, the SR-μCT technique may also be able to provide solution for estimating the age of the stone and the mineralization process.

## Supporting information

S1 Table The blood and urine parameters of the patients with kidney stone. (DOCX)

S2 Table Porosity value of pure and mixed types of kidney stones. (DOCX)

S1 Fig Micro-morphology of pure calcium oxalate monohydrate. (a & b) Different stones obtained from same patient (KS 8 and KS 16). (c & d) Different stones obtained from same patient (KS 1 and KS 2).
(TIF)

S2 Fig The tomographic slice image of different sections of the calcium oxalate monohydrate stone showing mineral precipitation and accumulation of minerals at different elevation plane. (i–xx) Different sections of the stones showing mineral precipitation and accumulation of minerals at different elevation plane. Arrows indicates the nucleus.
(TIF)

S3 Fig Micro-tomography of mixed kidney stones. (a) COM-COD mixed showing uniform micro-tomography and (b) COM-struvite-apatite mixed stones. Apatite showing comparatively denser structure (arrow).
(TIF)
