# Supplementary material for: Morphological characteristics and microstructure of kidney stones using synchrotron radiation μCT reveal the mechanism of crystal growth and aggregation in mixed stones
Source: PLoS One. 2019 Mar 22;14(3):e0214003. doi: 10.1371/journal.pone.0214003 (PMC6430423; doi:10.1371/journal.pone.0214003)
Supplement: S1 Table — (DOCX) [file pone.0214003.s001.docx]

**S1 Table. Blood and urine biochemistries in patients with kidney stone disease**

| Sl. No | Chemical composition | Hemoglobin  (g/dL) | RBC  (mcL) | Serum calcium  (mg/dL) | Serum uric acid  (mg/dL) | Random  blood sugar  (mg/dL) | Blood urea  (mmol/L) | Serum creatinine  (mg/dL) | ESR | Sodium  (mmol/L) | Potassium  (mmol/L) | Chloride  (mmol/L) | Urine pH |
| --- | --- | --- | --- | --- | --- | --- | --- | --- | --- | --- | --- | --- | --- |
| **KS-1, KS-2** | COM | 9.7 | 4.85 | 10.6 | 5.4 | 206 | 18 | 0.7 | 25 | 147 | 4.1 | 107 | 7.56 |
| **KS-3** | Uric acid | 12.9 | 4.81 | 9.3 | 6.8 | 148 | 24 | 1 | 45 | 146 | 4.5 | 105 | 7.9 |
| **KS-4** | Struvite- apatite mixed | 11.8 | 3.64 | 10 | 6.3 | 97 | 17 | 0.3 | 15 | 146 | 4 | 102 | 5.6 |
| **KS-5** | COM-uric acid mixed | 15.2 | 5.18 | 9.4 | 5.9 | 106 | 18 | 0.9 | 8 | 147 | 4.7 | 102 | 7.3 |
| **KS-6 , KS-8**  **KS-16** | COM | 12.6 | 4.45 | 10.3 | 2.3 | 107 | 27 | 0.8 | 20 | 149 | 2.8 | 109 | 6.5 |
| **KS-7** | Struvite - COM-apatite mixed | 12.9 | 4.69 | 8.1 | 5.2 | 103 | 19 | 0.6 | 5 | 140 | 4.8 | 98 | 6.7 |
| **KS-9** | COM-apatite mixed | 10.5 | 3.96 | 10 | 4.2 | 89 | 37 | 1.5 | 20 | 140 | 4.5 | 104 | 5.5 |
| **KS-10, KS-11** | Struvite | 12.2 | 4.45 | 7.2 | 5.5 | 81 | 18 | 0.7 | 5 | 140 | 5.3 | 100 | 6.3 |
| **KS-12** | COM-COD-apatite | 10.9 | 4.44 | 7.9 | 5.4 | 164 | 28 | 0.6 | 25 | 140 | 5.3 | 100 | 5.5 |
| **KS-13** | COM -uric acid mixed | 9.7 | 4.68 | 8.7 | 6.9 | 96 | 16 | 0.8 | 15 | 151 | 5.6 | 102 | 6.88 |
| **KS-14** | COM-COD mixed | 12.2 | 4.4 | 10.33 | 5.5 | 121 | 29 | 0.7 | 20 | 138 | 3.5 | 103 | 6.5 |
| **KS-15** | Struvite-apatite mixed | 12.1 | 4.36 | 9.3 | 5.4 | 115 | 42 | 1.2 | 25 | 139 | 5.7 | 98 | 5.71 |
| **KS-17** | Uric acid | 12.9 | 4.71 | 9.4 | 6.7 | 123 | 25 | 0.7 | 35 | 139 | 5.3 | 109 | 6.78 |
| **KS-18, KS-19** | COM-COD-apatite mixed | 12.2 | 4.13 | 10.7 | 4.2 | 119 | 19 | 0.7 | 5 | 139 | 4.1 | 104 | 7.15 |
| **KS-20** | COM-COD-apatite mixed | 11.8 | 3.96 | 8.7 | 5.5 | 70 | 22 | 0.1 | 13 | 138 | 4.2 | 99 | 7.7 |
| **KS-21** | Struvite | 11.1 | 3.98 | 8.8 | 7.9 | 123 | 36 | 1.2 | 30 | 137 | 4.4 | 103 | 5.7 |
| **KS-22** | COM-apatite mixed | 11.9 | 4.56 | 9.3 | 5.8 | 126 | 26 | 1 | 18 | 139 | 4.3 | 106 | 5.8 |
