# Supplementary material for: Morphological characteristics and microstructure of kidney stones using synchrotron radiation μCT reveal the mechanism of crystal growth and aggregation in mixed stones
Source: PLoS One. 2019 Mar 22;14(3):e0214003. doi: 10.1371/journal.pone.0214003 (PMC6430423; doi:10.1371/journal.pone.0214003)
Supplement: S2 Table — (DOCX) [file pone.0214003.s002.docx]

**S2 Table . Mean porosity of pure and mixed types of kidney stones.**

| Sample | Total sample volume with including pores (μm^3^) | Total sample volume without pores (μm^3^) | Pore volume (μm^3^) | Pore volume fraction = pore volume/ total volume including pores | Porosity (%) |
| --- | --- | --- | --- | --- | --- |
| COM | 58274194105 | 58139811247 | 134382858 | 0.00230 | 0.23 |
| Uric acid | 114979005758 | 109963901926 | 5015103832 | 0.04361 | 4.36 |
| Struvite | 45302343592 | 41719652316 | 3582691276 | 0.07908 | 7.90 |
| COM-apatite | 86954057300 | 80370622416 | 6583434884 | 0.07570 | 7.57 |
| COM-uric acid | 108873314463 | 105733243920 | 3140070543 | 0.02884 | 2.88 |
| Struvite-apatite | 111763548295 | 109068536542 | 2695011753 | 0.02411 | 2.41 |
| COM-COD | 34087352553 | 30415499291 | 3671853262 | 0.1077 | 10.77 |
| COM-COD-apatite | 66101941775 | 61121146687 | 4980795088 | 0.07535 | 7.53 |
