# Supplementary figures and images for: Morphological characteristics and microstructure of kidney stones using synchrotron radiation μCT reveal the mechanism of crystal growth and aggregation in mixed stones
Source: PLoS One. 2019 Mar 22;14(3):e0214003. doi: 10.1371/journal.pone.0214003 (PMC6430423; doi:10.1371/journal.pone.0214003)

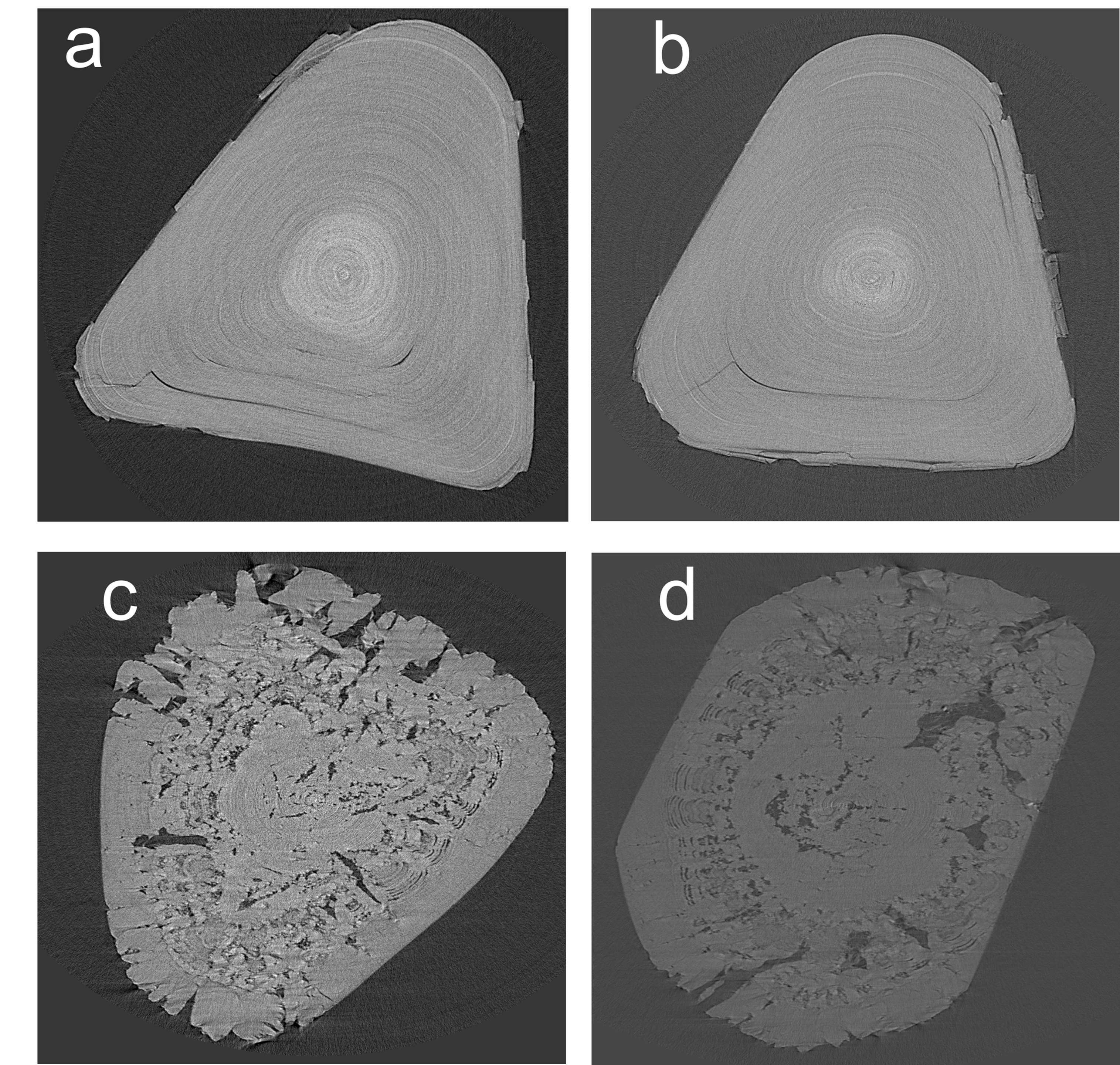

Supplement: S1 Fig — (a & b) Different stones obtained from same patient (KS 8 and KS 16). (c & d) Different stones obtained from same patient (KS 1 and KS 2). (TIF) [file pone.0214003.s003.tif]

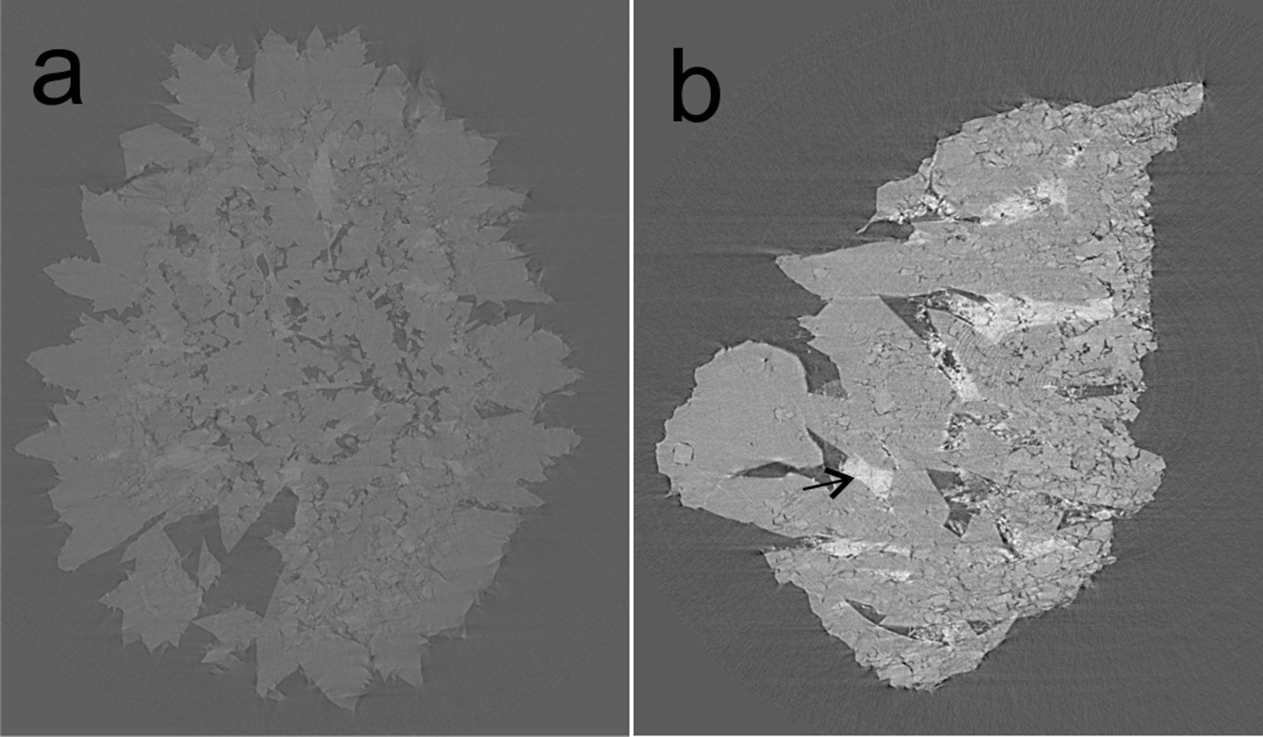

Supplement: S3 Fig — (a) COM-COD mixed showing uniform micro-tomography and (b) COM-struvite-apatite mixed stones. Apatite showing comparatively denser structure (arrow). (TIF) [file pone.0214003.s005.tif]
